# Limited Evidence That Developmental Diet and Density Affect Adult Female Earwig Behaviour Despite Repeatable Individual Differences

**DOI:** 10.1002/ece3.74325

**Published:** 2026-09-02

**Authors:** Clint D. Kelly, Jules Vaillancourt, Clara Smythe, Céline Ozkan

**Affiliations:** ^1^ Département des Sciences biologiques Université du Québec à Montréal Montréal Quebec Canada; ^2^ Département de biologie, chimie et géographie Université du Québec à Rimouski Rimouski Quebec Canada; ^3^ Université Claude Bernard Lyon 1, LBBE, UMR 5558, CNRS, VetAgro Sup Villeurbanne France

## Abstract

Developmental environments can have lasting effects on adult behaviour, shaping mean activity, repeatability and within‐individual variability in potentially independent ways. We used a split‐brood experiment to test whether developmental diet quality and social density affected adult open‐field behaviour in female European earwigs (*Forficula auricularia sensu lato*). Nymphs were reared on a standard or nutrient‐diluted ad libitum diet at one, four or eight individuals per dish. Adult females were assayed six times over 16–21 days. Females showed repeatable individual differences in open‐field behaviour, particularly distance travelled. A beta‐binomial mixed‐effects analysis showed substantial treatment‐associated differences in survival, which was lower on the nutrient‐diluted diet and lower in group‐reared than individually reared nymphs. These differences confirmed biological consequences of the treatments and indicated treatment‐dependent selective survival that may have filtered phenotypes before testing. Multivariate Bayesian mixed‐effects models provided limited evidence that developmental diet or density consistently affected mean behaviour, repeatability, individual change across trials or residual within‐individual variability among surviving adult females. Distance travelled and centre use were strongly negatively correlated among individuals; because both were derived from the same arena assay, we interpret this as a shared axis of arena use rather than an independent behavioural syndrome. Repeatable individual differences in open‐field behaviour were thus detectable but not clearly explained by the developmental treatments, with treatment‐dependent selective survival an additional interpretive constraint.

## Graphical abstract

We used a split‐brood experiment to test whether developmental diet quality and social density shape adult open‐field behaviour in female European earwigs across six repeated trials. Developmental treatments had little consistent effect on mean behaviour or behavioural variance, whereas individuals showed repeatable differences in distance travelled and weaker repeatability in centre use; these measures were strongly negatively correlated at the among‐individual level. Our results indicate that stable individual differences in adult behaviour can emerge even when they are only weakly explained by ecologically relevant developmental conditions.

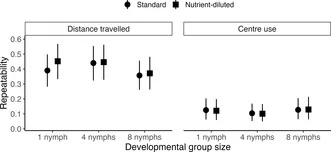

## 1 Introduction

Individuals within populations often differ consistently in behaviour. These repeatable among‐individual differences, commonly termed animal personality, occur across diverse taxa and can influence movement, resource acquisition, social interactions and responses to environmental change (Dingemanse et al. 2010; Laskowski et al. 2022; Réale et al. 2007; Sih et al. 2004). Behavioural variation also includes differences in how individuals change across repeated experiences and how variable they are around their own expected behaviour. Distinguishing mean behaviour, behavioural plasticity and behavioural predictability can therefore reveal developmental effects that would be missed by comparing treatment means alone (Biro and Adriaenssens 2013; O'Dea et al. 2022; Stamps et al. 2012; Stamps 2016).

Conditions experienced during development can shape adult behavioural phenotypes by altering growth, energetic state, physiology and social experience (Kasumovic and Brooks 2011; Stamps and Groothuis 2010; West‐Eberhard 2003). Experimental studies show that developmental nutrition, social experience, physical complexity, predation risk and incubation conditions can influence adult behavioural means, among‐individual variation and repeatability (De Jong et al. 2022; DiRienzo et al. 2019; Kaiser et al. 2019; Noguera et al. 2015; Tariel et al. 2020; Xu et al. 2021). These components need not respond together: developmental conditions can alter behavioural means without changing repeatability (Xu et al. 2021), alter repeatability through changes in among‐individual variation (DiRienzo et al. 2019; Kaiser et al. 2019), or affect within‐individual variation (De Jong et al. 2022). Far less is known about whether developmental conditions affect individual behavioural change across repeated experiences or behavioural predictability. Consequently, weak treatment effects on behavioural means do not necessarily imply that development was unaffected, and effects on adult behaviour must be evaluated alongside changes in variance components, the biological consequences of the manipulation and its possible selective effects.

The European earwig, *Forficula auricularia sensu lato*, is well suited to this question because its development occurs in a social and nutritionally variable setting. Females care for eggs and young nymphs, while sibling interactions include both food sharing and competition (Falk et al. 2014; Kölliker 2007; Kramer et al. 2015; Meunier 2024). Maternal and sibling environments can have persistent consequences for development and later phenotypes (Cheutin et al. 2024; Thesing et al. 2015; Van Meyel and Meunier 2022), and maternal nutritional experience can alter offspring performance under different food environments (Raveh et al. 2016). Nutrition and density also influence body‐size and male forceps expression (Ozkan and Kelly 2026; Tomkins 1999; Tomkins and Brown 2004). These findings establish diet composition and social conditions as biologically relevant developmental variables, but do not establish that adult open‐field behaviour will also be affected.

Adult activity can affect movement and encounter rates with food, shelters and conspecifics, making persistent developmental effects potentially relevant to the ecological performance of earwigs as omnivorous consumers. Open‐field assays, however, do not isolate a single ecological trait. Distance travelled may reflect locomotor activity, responses to novelty or disturbance, and centre use can partly arise from arena geometry. We therefore treat the two responses as measures of open‐field activity and spatial arena use, not as definitive measures of exploration, boldness or a behavioural syndrome.

We manipulated developmental diet composition and post‐family social density using a split‐brood design. Offspring from maternal families were distributed across two ad libitum diets and densities of one, four or eight nymphs per dish where family size permitted; maternal family was retained in the statistical model rather than assumed to have been eliminated by the design. We then measured distance travelled and centre use in adult females across six repeated open‐field trials. We predicted that females reared on the nutrient‐diluted diet would travel less and use the centre less if developmental nutritional limitation reduced adult energetic state or locomotor capacity (Kasumovic and Brooks 2011; Noguera et al. 2015). We did not make a single directional prediction for density because isolation, social stimulation and competition could have opposing effects. Instead, we predicted that isolation versus group rearing, and potentially group size, could alter (1) mean adult open‐field behaviour, (2) repeatable among‐individual variation, if developmental conditions amplify or buffer individual differences (Stamps et al. 2012), (3) individual change across trials, if developmental background shapes sensitivity to repeated exposure, and/or (4) residual within‐individual variability, if more variable or stressful rearing increases behavioural unpredictability (Biro and Adriaenssens 2013). We also quantified survival to adulthood because treatment‐dependent attrition could both validate the biological effect of the manipulation and selectively filter the adults available for behavioural testing.

## 2 Methods

### 2.1 Ethics Declaration

No permits or institutional animal ethics approval were required for this study under Canadian law because the research involved an invertebrate species. Earwigs were maintained under controlled laboratory conditions with access to food, water and moist substrate. Adult experimental animals were euthanized by freezing at −20°C after the experiment. All efforts were made to minimise disturbance and unnecessary harm.

### 2.2 Study Animals and Laboratory Maintenance

Adult female earwigs morphologically assignable to the *Forficula auricularia* species complex were collected by hand at night from vegetation in Parc Outremont, Montréal, Québec, Canada, during September–October 2024. Because individuals were not genetically assigned to lineage, we refer to them as *F. auricularia sensu lato* (González‐Miguéns et al. 2020).

A total of 115 adult females were transported to the Université du Québec à Montréal and housed individually in 90‐mm Petri dishes containing moist sand, a cotton‐plugged water tube and TetraMin Tropical Flakes. Earwigs were maintained in darkness at 10°C in a Percival incubator. Dishes were checked daily for oviposition, after which food and water were removed. Beginning in January 2025, dishes were checked daily for hatching. Hatching occurred from 13 to 29 January 2025, and all 115 females produced offspring. Families received TetraMin Tropical Flakes and water ad libitum for 2 weeks after hatching, allowing nymphs to complete an early moult before transfer to their experimental dishes.

### 2.3 Developmental Environment Manipulation

We manipulated diet composition and post‐family social density. The standard diet consisted of 100% TetraMin Tropical Flakes; the nutrient‐diluted diet consisted of 75% TetraMin Tropical Flakes and 25% α‐cellulose. Both diets were available ad libitum. The manipulation therefore reduced nutrient concentration rather than directly controlling food intake, and nymphs may have partly compensated by increasing consumption. Group‐size treatments contained one, four, or eight nymphs per 90‐mm dish. These levels were not intended to reproduce natural brood sizes. Rather, they contrasted individual rearing with progressively larger post‐family social groups, thereby changing opportunities for contact, competition, aggregation and other social interactions under standardised space and food availability. Because dish area was held constant, group size, numerical density and per‐capita space covaried; the experiment cannot isolate social contact from crowding or other consequences of increased occupancy.

Offspring from each maternal family were randomly distributed across diet and density combinations where family size permitted. Assignment occurred after the initial two‐week family period; thus, all offspring experienced the egg environment, maternal care and early sibling environment before treatment. The split‐brood allocation distributed maternal backgrounds across treatments, and maternal family was included as a random effect, but the design did not remove maternal or early‐family effects. Experimental dishes were maintained at 20°C and contained a water tube, the assigned ad libitum diet and a refuge made from four pieces of paper straw. Dishes were checked several times per week for mortality and adult emergence.

Upon adult emergence, individuals were removed from their developmental dishes, sexed from forceps morphology and assigned unique adult identifiers. Females were isolated on the day of adult emergence in individual Petri dishes maintained at 20°C with a refuge, a water tube and 100% fish flakes provided ad libitum. The common adult diet and housing conditions were maintained throughout the 16–21‐day testing period so that the experiment assessed persistent effects of developmental conditions rather than combined developmental and adult environments. The first behavioural assay occurred approximately 1 week after adult emergence. Consequently, females reared in four‐ or eight‐nymph groups experienced a transition to individual housing before testing, whereas one‐nymph females remained isolated.

### 2.4 Behavioural Assays

We randomly selected 160 adult females across the developmental treatments and allocated them across morning testing blocks. One female escaped after the first trial and was excluded from analysis. The remaining 159 females each completed six trials, producing 954 observations. Earwigs were maintained on a reversed light cycle so that assays conducted in the morning occurred during their subjective night. Trials were conducted in darkness between 08:30 and 12:00 h, with two blocks tested per day and block order balanced across the morning where possible.

Open‐field arenas were clear 90‐mm Petri dishes placed on an infrared backlight array and filmed from above with Basler Ace monochrome infrared‐sensitive cameras. Cameras were connected to a computer running EthoVision XT (Noldus et al. 2001). The apparatus was enclosed within a Percival incubator maintained at 20°C in darkness.

Before every trial, females were exposed briefly to CO_2_ to permit standardised placement in the centre of the arena. The effect lasted approximately 20–30 s. Females typically resumed walking about 5 s after placement, and recording began when movement resumed; no individual remained stationary in the arena centre after recovery. Each trial lasted 10 min, after which arenas were cleaned with 95% ethanol. Trials were separated by 2 to 3 days. Because all females received the same procedure, CO_2_ exposure was standardised across treatments, although the design did not include a non‐anaesthetized control.

EthoVision XT quantified distance travelled (cm) and time spent in a circular centre zone 25 mm in diameter. Distance travelled was interpreted as locomotor activity in the open field. Because individuals began each trial in the centre, centre use was calculated only after the first exit; time before that exit was excluded. We interpret centre use as spatial behaviour within this arena rather than a direct measure of boldness.

### 2.5 Morphological Measurements

Pronotum length was measured as an index of adult body‐size. Adults were photographed using a Leica S6D stereomicroscope at 0.68× magnification with Enersight software, and pronotum length was measured to the nearest 0.001 mm in Fiji (Schindelin et al. 2012). A subset of 30 individuals was measured twice; repeatability was high (intraclass correlation coefficient = 0.94).

### 2.6 Statistical Analyses

Analyses were conducted using R 4.4.3 (R Core Team 2025). Distance travelled and centre use were log‐transformed after adding one to reduce right skew and then standardised. Standardisation placed coefficients on response‐standard‐deviation scales and facilitated comparison between behaviours. Trial number was centred and standardised so that model intercepts represented behaviour near the middle of the six‐trial sequence and to improve estimation of random intercept–slope covariance. Pronotum length was standardised for the body‐size sensitivity analysis.

We reconstructed survival to adulthood for all assigned nymphs. For each developmental dish, the number recorded as surviving to adulthood was the number with an adult identifier; individuals without an adult identifier—including recorded deaths, disappearances, and unresolved absences—were classified as not having been recorded as surviving to adulthood. Because nymphs without adult identifiers could not be sexed, survival was analysed for both sexes combined. Survival was analysed at the developmental‐dish level for all 1047 dishes across the 115 maternal families; the 159 assayed females were drawn from 126 of these dishes. We fitted a Bayesian beta‐binomial mixed‐effects model in *brms* to account for extra‐binomial variation across dishes. The number reaching adulthood was specified as successes out of the number initially assigned to each dish. Diet, density and their interaction were treated as fixed effects, with density coded as a three‐level factor. Maternal family was included as a random intercept to account for baseline differences among families. This specification does not eliminate within‐family treatment contrasts generated by the split‐brood design; the family random intercept absorbs family‐level variance around the treatment means without conflating the treatment estimates themselves. We derived population‐level posterior survival probabilities for each diet–density combination and probability‐scale contrasts between diets within density and between densities within diet. The model used the same chain settings and convergence criteria described below for the behavioural models. Raw counts and observed proportions are reported alongside model‐estimated survival probabilities and 95% credible intervals. A posterior predictive check for the survival model is shown in Figure S1.

Adult behaviour was analysed with a multivariate Bayesian mixed‐effects model in *brms* (Bürkner 2017). Distance travelled and centre use were modelled jointly. For each response, the mean model included diet, density, centred trial number, all interactions among them, and testing block. The interactions were retained because the hypotheses included treatment‐specific change across repeated trials; weakly informative priors regularised these estimates. Individual identity had correlated random intercepts and trial slopes across responses, maternal family had a random intercept, and developmental dish had a random intercept to account for shared rearing environment among the 159 females distributed across 126 dishes. Among‐individual variance (individual random intercept) was assumed to be constant across treatments. Treatment‐specific repeatabilities therefore differ only through treatment‐dependent residual variance variation, not through changes in among‐individual variance itself. This model can assess whether treatments alter mean behaviour, trial trajectories, or residual within‐individual variability, but cannot directly estimate whether treatments change the among‐individual variance. The residual standard deviation was modelled as a function of diet, density and their interaction, with an individual random intercept to estimate consistent differences in residual within‐individual variability. Because both responses were measured simultaneously within the same arena trial, residual correlations were estimated to account for potential within‐trial covariance arising from shared arena conditions, transient behavioural states, and trajectory geometry. A sensitivity analysis with residual correlations fixed to zero evaluated whether this specification substantively affected the among‐individual correlation estimates.

A secondary model added standardised pronotum length to both mean submodels. Models used four chains of 4000 iterations, including 1000 warm‐up iterations, with adapt_delta = 0.97 and maximum tree depth 12. Convergence was assessed with trace plots, effective sample sizes and *R̂*. Posterior means and 95% credible intervals describe effect magnitude and precision; we do not treat intervals overlapping zero as proof of no effect and do not use retrospective power calculations. Repeatability was calculated from posterior draws as individual‐intercept variance divided by individual‐intercept plus residual variance for each treatment, evaluated at the centred trial (approximately the middle trial, Trial 3–4) because the model includes random trial slopes. This calculation used population‐level residual standard deviations and did not integrate over the individual random effect in the sigma model, so repeatabilities represent values for an individual with average log‐residual‐SD within each treatment. Family and individual variance proportions, random trial‐slope variation, individual variation in residual standard deviation and among‐individual correlations were also derived from posterior draws. Because distance and centre use were measured in one arena, their correlation was interpreted as shared arena use rather than an independent behavioural syndrome. Posterior predictive checks for the primary behavioural model are shown in the Figures S2 and S3.

### 2.7 Prior Specification and Model Diagnostics

The behavioural models used Bayesian inference with weakly informative priors. Population‐level coefficients (fixed effects) received *Normal* (0, 1) priors, which place 95% probability mass between ±2 units on the standardised scale. Intercepts received *Normal* (0, 2) priors. Group‐level standard deviations received *Student‐t* (ν = 3, *μ* = 0, *σ* = 1) priors. Correlations among random effects used an LKJ (*η* = 2) prior. In the sigma submodels, fixed effects for diet and density received *Normal* (0, 1) priors and the intercept received *Normal* (0, 1). The survival model used the same prior structure on coefficients but with a *Normal* (0, 2) intercept prior. Residual correlations between distance travelled and centre use were estimated by default in *brms*.

Primary behaviour model: all parameters had $\hat{R}\leq 1.001$, with minimum effective sample sizes of 3074 iterations (bulk) and 5674 iterations (tail) out of 12,000 total post‐warmup draws. Secondary (size‐adjusted) behaviour model: maximum $\hat{R}=1.002$, with minimum effective sample sizes of 3460 (bulk) and 4954 (tail). Beta‐binomial survival model: maximum $\hat{R}=1.001$, minimum effective sample sizes 5392 (bulk) and 7617 (tail). No divergent transitions or maximum tree‐depth warnings were detected in any model.

## 3 Results

### 3.1 Developmental Survival Varied Among Treatments

Survival before adulthood varied markedly across developmental environments (Table 1). Nymphs reared on the nutrient‐diluted diet consistently showed lower survival than those reared on the standard diet (risk differences: −26.9 percentage points, 95% CrI [−33.8, −20.0] at one nymph per dish, −19.7 percentage points, 95% CrI [−24.8, −14.5] at four nymphs per dish, −29.1 percentage points, 95% CrI [−33.0, −25.2] at eight nymphs per dish), and survival was lower at four or eight nymphs per dish than at one nymph, with reductions of −14.2 percentage points, 95% CrI [−18.1, −10.1] on the standard diet and −16.5 percentage points, 95% CrI [−23.5, −9.2] on the nutrient‐diluted diet. Risk differences for diet are reported as nutrient‐diluted minus standard diet; risk differences for group size are reported as larger minus smaller group size. These treatment‐associated differences demonstrated that the treatment combinations were associated with biologically consequential differences in attrition and indicated treatment‐dependent selective survival that may have filtered phenotypes before testing. The behavioural sample nevertheless represents survivors from environments with unequal attrition, creating the possibility of treatment‐dependent selective filtering.

**TABLE 1:** Survival to adulthood across developmental diet and density treatments.

Diet	Density	Dishes	Assigned	Survived	Not recorded as surviving	Observed survival (%)	Modelled survival (95% CrI)
Standard	1	253	253	235	18	92.9	90.8% [87.4, 93.8]
Standard	4	155	620	465	155	75.0	75.3% [71.9, 78.7]
Standard	8	144	1152	881	271	76.5	76.6% [74.2, 79.0]
Nutrient‐diluted	1	197	197	123	74	62.4	64.0% [57.3, 70.2]
Nutrient‐diluted	4	169	676	377	299	55.8	55.6% [51.9, 59.3]
Nutrient‐diluted	8	129	1032	490	542	47.5	47.5% [44.4, 50.5]

*Note:* Counts include both sexes because individuals not recorded as reaching adulthood could not be sexed. Observed survival rates are shown alongside modelled estimates from a beta‐binomial mixed‐effects model with maternal family random intercept and 95% credible intervals.

### 3.2 Model Performance and Analytical Sample

All three models converged well (survival model: maximum *R̂* = 1.00; behavioural models: maximum *R̂* = 1.00 for both the primary and body‐size‐adjusted models). The primary model included 954 observations from 159 females belonging to 72 maternal families. A maternal family contributed a median of 1.5 analysed females (range 1–10). Final sample sizes were standard‐1 = 30, standard‐4 = 25, standard‐8 = 37, nutrient‐diluted‐1 = 22, nutrient‐diluted‐4 = 22, nutrient‐diluted‐8 = 23 females. The body‐size‐adjusted model gave qualitatively similar estimates. Pronotum length was not clearly associated with distance travelled (*β* = 0.01, 95% CrI [−0.13, 0.14]) or centre use (*β* = −0.00, 95% CrI [−0.10, 0.09]), so we report the primary model below.

### 3.3 Limited Evidence for Effects on Mean Open‐Field Behaviour

Developmental diet and group size provided limited evidence of consistent effects on mean distance travelled. At one‐nymph density (the reference level), nymphs reared on the nutrient‐diluted diet did not differ from standard‐diet nymphs (*β* = 0.06, 95% CrI [−0.32, 0.43]). Under the standard diet (the reference level), the effects of four‐nymph (*β* = −0.14, 95% CrI [−0.51, 0.23]) and eight‐nymph (*β* = −0.03, 95% CrI [−0.37, 0.32]) groups relative to one nymph were imprecise. Other diet, density and trial interactions also had intervals spanning zero. Raw‐scale median and interquartile‐range summaries of distance travelled, and centre use by treatment are provided in Table S1 for biological context.

Effects on centre use were similarly uncertain. Main effects of nutrient‐diluted diet (*β* = −0.03, 95% CrI [−0.31, 0.24]), four‐nymph group (*β* = −0.03, 95% CrI [−0.30, 0.24]) and eight‐nymph group (*β* = 0.18, 95% CrI [−0.07, 0.42]) were imprecise. Some trial‐related interactions were estimated away from zero: the trial effect was more positive for nutrient‐diluted than standard‐diet nymphs (*β* = 0.26, 95% CrI [0.05, 0.46]). For four‐nymph groups, the trial slope was more positive than for one‐nymph groups (*β* = 0.21, 95% CrI [0.01, 0.42]), and for eight‐nymph groups it was similarly elevated (*β* = 0.16, 95% CrI [−0.02, 0.33]). The nutrient‐diluted × four‐nymph × trial interaction was negative (*β* = −0.37, 95% CrI [−0.68, −0.06]). Because these terms did not form a simple directional pattern and arose within a parameter‐rich interaction structure, we interpret them cautiously (see Figure S4).

### 3.4 Repeatable Individual Differences

**FIGURE 1:**
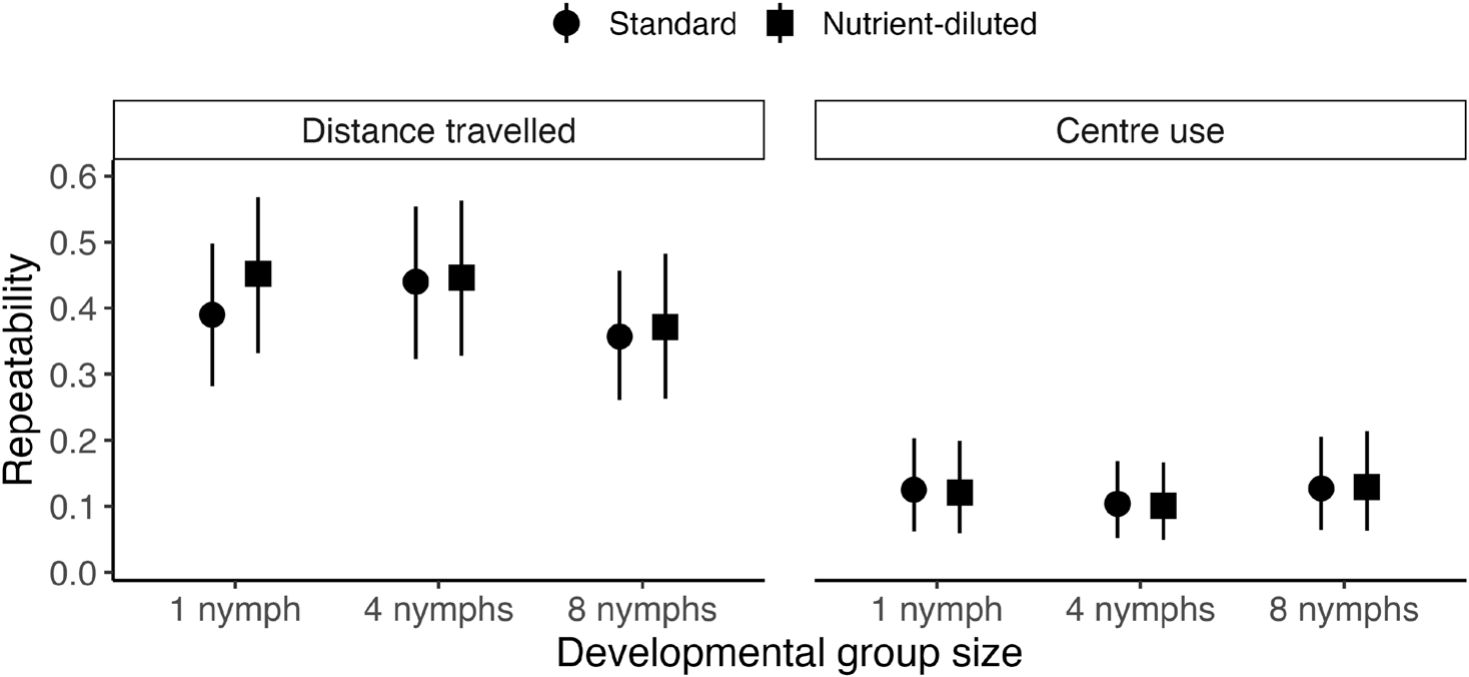
Repeatability of distance travelled and centre use across developmental diet and group‐size treatments. Points show posterior means and vertical lines show 95% credible intervals. Among‐individual variance was assumed constant across treatments; consequently, treatment‐specific repeatabilities differ only through treatment‐dependent residual variance. Credible intervals overlap broadly, providing limited evidence that diet or group size altered repeatability through residual‐variance differences.

Individual intercept variation was stronger for distance travelled (SD = 0.603, 95% CrI [0.491, 0.713]) than for centre use (SD = 0.319, 95% CrI [0.211, 0.421]). Among‐individual variance was assumed constant across treatments; treatment‐specific posterior mean repeatabilities therefore ranged from 0.34 to 0.45 for distance travelled and 0.10 to 0.12 for centre use (Figure 1) only because residual variance differed among treatments. Credible intervals overlapped broadly among treatment combinations, providing limited evidence that diet or density altered repeatability through changes in residual variance.

When maternal‐family variance was included in the denominator, individual identity accounted for 0.34 to 0.43 of the modelled variance (individual plus family components) in distance travelled and 0.10 to 0.12 in centre use. Maternal family accounted for only 0.022 to 0.028 and 0.014 to 0.018 of this modelled variance, respectively, with substantial uncertainty. This variance accounting does not include the developmental‐dish random effect. Variance components from the fitted model indicated posterior mean dish random‐effect SDs of SD = 0.159, 95% CrI [0.009, 0.385] for distance and SD = 0.105, 95% CrI [0.005, 0.256] for centre, but their lower credible limits were close to zero (0.009 and 0.005, respectively), so the evidence that dish‐level variance is meaningful is weak. The point estimates are similar in magnitude to maternal‐family effects, suggesting that shared rearing containers did not substantially constrain individual behavioural variation.

### 3.5 Weakly Supported Variation in Change and Predictability

To assess the robustness of our linear characterisation of trial effects, we performed a sensitivity analysis by refitting the primary behavioural model with trial treated as a factor rather than a continuous variable. A sensitivity model treating trial as a factor produced no clear departures from the conclusions of the linear‐trial model, supporting the adequacy of the linear specification.

Random trial‐slope standard deviations were small: SD = 0.111, 95% CrI [0.013, 0.202] for distance and SD = 0.057, 95% CrI [0.002, 0.159] for centre use. Raw trajectories of repeated individual observations across trials are shown in Figures S5 and S6. Correlations between individual intercepts and slopes were imprecise for distance (*r* = 0.37, 95% CrI [−0.16, 0.78]) and centre use (*r* = 0.12, 95% CrI [−0.58, 0.75]), as was the correlation between their trial slopes (r = −0.04, 95% CrI [−0.72, 0.67]). These estimates provide, at most, weak support for stable individual differences in change across trials.

Individuals differed modestly in residual variability on the log‐sigma scale for distance (SD = 0.142, 95% CrI [0.015, 0.257]) and centre use (SD = 0.186, 95% CrI [0.063, 0.284]). Treatment effects in the sigma submodels were imprecise, providing limited evidence that the developmental environment explained behavioural predictability.

### 3.6 Correlated Measures of Arena Use

Distance travelled and centre use were strongly negatively correlated at the among‐individual level (r = −0.66, 95% CrI [−0.87, −0.42]). Females that consistently travelled farther tended to spend less time in the centre. Because both measures were generated simultaneously in the same arena trial, we estimated residual correlations to account for potential within‐trial covariance. The residual correlation between distance travelled and centre use was *r* = −0.23, 95% CrI [−0.30, −0.16], indicating modest shared variance from transient trial‐level states, arena conditions, or trajectory geometry beyond the among‐individual relationship. A sensitivity analysis that fixed residual correlations to zero yielded an among‐individual correlation of *r* = −0.74, 95% CrI [−0.91, −0.52] compared to *r* = −0.66, 95% CrI [−0.87, −0.42] in the main model, indicating the among‐individual correlation was robust to the residual‐correlation specification. The relationship likely reflects a shared axis of movement and spatial use, including perimeter following, and is not sufficient evidence for a syndrome across independent behaviours.

## 4 Discussion

Adult female earwigs showed repeatable individual differences in open‐field behaviour, especially distance travelled, demonstrating consistent differences among individuals within this assay (Réale et al. 2007; Sih et al. 2004). The developmental treatments did not leave clear, consistent signatures on mean behaviour, repeatability or residual variability among the surviving females, though this conclusion is narrower than claiming that developmental environment does not affect earwig personality generally: it applies to two correlated open‐field measures derived from the same arena trial, to females, and to survivors of the particular diet and density treatments used here.

The formal survival analysis changes how the weak adult behavioural effects should be interpreted. Both the raw proportions and model‐estimated probabilities showed substantially lower survival on the nutrient‐diluted diet and lower survival in group‐reared than individually reared nymphs; raw attrition reached 52.5% under the nutrient‐diluted diet at eight nymphs per dish. The manipulation was therefore biologically consequential, even though food was supplied ad libitum. However, ad libitum access means that we manipulated nutrient concentration rather than measured nutrient intake. Compensatory feeding—an increase in consumption when food is nutrient‐diluted—is well documented in insects (Rho and Lee 2023; Timmins et al. 1988; Wheeler and Slansky Jr 1991). Under moderate dilution, increased consumption and nutrient utilisation can maintain growth or pupal mass close to that observed on an undiluted diet, although costs can remain in other traits such as lipid accumulation (Wheeler and Slansky Jr 1991). Compensation is also frequently incomplete, particularly when cellulose is used as the diluent (Rho and Lee 2023; Timmins et al. 1988). Thus, increased consumption by earwig nymphs need not have eliminated all nutritional consequences of the diluted diet, as indicated by their lower survival, but it could have reduced the realised difference in nutrient intake among surviving individuals and thereby attenuated adult behavioural differences. More importantly, unequal attrition creates selective filtering. Individuals most susceptible to poor diet or group rearing may have died before adulthood, potentially reducing phenotypic differences among the survivors. Because unequal attrition created selective filtering, we cannot determine whether the weak adult behavioural treatment effect reflects true buffering of behaviour or convergence of survivors to similar phenotypes.

The group‐size treatment should also be interpreted as a controlled contrast in post‐family social conditions, not natural brood density. One‐nymph groups developed alone, whereas four‐ and eight‐nymph groups experienced progressively more conspecific contact within the same dish area. The primary expected mechanism was developmental cost through resource competition or differential social stimulation (Kölliker 2007; Kramer et al. 2015), but ad libitum food availability likely attenuated competitive interactions, leaving variation in social contact and aggregation as the main contrast between group sizes. Upon adult emergence, all females were isolated for approximately 1 week before testing. Thus, group‐reared females experienced a social transition that one‐nymph females did not. Social isolation can alter physiological responses in adult earwigs (Kohlmeier et al. 2016), so this common adult housing step may have affected group‐reared females or attenuated differences generated during development. The one‐week interval reduced the likelihood that we measured only an immediate handling response, but it does not eliminate longer‐lasting effects of isolation. Standardising adult conditions was nevertheless necessary to separate persistent developmental effects from continued adult treatment exposure.

We found limited evidence for treatment effects on any of our four predictions: mean behaviour (Prediction 1), repeatability (Prediction 2), trial slopes (Prediction 3), and residual variability (Prediction 4). The clearest positive finding was consistent among‐individual variation in open‐field behaviour (Laskowski et al. 2022; Réale et al. 2007; Sih et al. 2004). Distance travelled was moderately repeatable and centre use less so, and this consistency was present across all treatment groups. Importantly, the posterior estimates supported among‐individual variation in open‐field behaviour: posterior mean repeatability for distance travelled was 0.34 to 0.45 across treatments, with credible intervals excluding zero in all cells. Because the model assumed constant among‐individual variance across treatments, these treatment‐specific repeatability estimates reflect differences in residual variance, not differences in among‐individual variance. Developmental diet and density did not, however, clearly alter repeatability through residual variance changes, as credible intervals for treatment‐specific repeatability overlapped broadly. Maternal‐family variance was small in this dataset, but the split‐brood design does not imply that maternal effects were absent. All offspring shared prenatal and early post‐hatching influences (Cheutin et al. 2024; Thesing et al. 2015; Van Meyel and Meunier 2022), and maternal food environments can program offspring performance in this species (Raveh et al. 2016). The family estimates simply indicate that, after accounting for the modelled sources of variation, the two adult female open‐field responses were not strongly clustered by maternal identity. Notably, developmental effects on different behavioural components show variable concordance in other taxa: some environmental conditions alter mean behaviour without affecting repeatability (Xu et al. 2021), whereas others alter within‐individual variability (De Jong et al. 2022). Our findings add to this variation: we detected neither clear mean effects nor residual‐variability effects, suggesting either buffering or selective filtering of behavioural responses in earwigs.

Evidence for individual differences in trial slopes and residual predictability was similarly modest. Random trial‐slope standard deviations were estimated with lower credible limits extremely close to zero, and their correlations were imprecise, so strong claims about individual variation in habituation or sensitization (Stamps et al. 2012; Stamps 2016) are not warranted. Treatment effects on residual variability were also imprecise. The data therefore support repeatable differences in average open‐field behaviour more strongly than differences in behavioural change or predictability.

The strong among‐individual negative correlation between distance travelled and centre use (*r* = −0.66, 95% CrI [−0.87, −0.42]) should not be interpreted as an independent behavioural syndrome (Dingemanse et al. 2010; Sih et al. 2004). The much weaker residual (within‐trial) correlation (*r* = −0.23, 95% CrI [−0.30, −0.16]) indicates that the strong among‐individual relationship reflects consistent individual differences in arena use strategy rather than shared trial‐level constraints. The among‐individual correlation was robust to whether residual correlations were estimated or fixed to zero (sensitivity model: *r* = −0.74, 95% CrI [−0.91, −0.52]), supporting the interpretation that the relationship between distance and centre use reflects consistent individual differences in arena use rather than trial‐level mechanical coupling. Both responses were extracted from the same trajectories, and the modest residual correlation likely reflects the mechanical consequence that greater perimeter movement inherently produces longer distances and less centre occupancy. Independent assays of shelter emergence, aggregation, foraging, disturbance responses or predator avoidance would be needed to test whether the observed axis of arena use generalises across contexts as a true behavioural syndrome.

Two procedural limitations further constrain inference. First, every trial followed brief CO_2_ anaesthesia. Without a non‐anaesthetized control, we cannot exclude effects of repeated exposure on locomotion or measurement noise (Bartholomew et al. 2015); however, standardisation across treatments makes this unlikely to generate spurious differences. More importantly, such effects could reduce the sensitivity of the experiment to detect genuine treatment differences if all groups were affected similarly. Second, only females were tested, which avoided confounding with male forceps morphs but precludes species‐wide comparisons between behavioural and morphological plasticity (Ozkan and Kelly 2026; Tomkins 1999; Tomkins and Brown 2004). Sample size limitations also constrain precision: small developmental effects would not be clearly resolved at this scale, and null results should be interpreted alongside this constraint rather than as evidence for developmental buffering.

In conclusion, developmental diet and post‐family density produced large differences in the proportion reaching adulthood but provided limited evidence of persistent effects on two adult female open‐field responses among survivors. Adult females nevertheless differed repeatably in arena behaviour. The combination of unequal survival, common adult conditions, pre‐test isolation and standardised CO_2_ exposure means that the adult results should be interpreted as conditional outcomes of the complete experimental sequence. Future experiments that follow behaviour before and after social transitions, include independent behavioural contexts and explicitly link survival with behavioural phenotypes will be needed to determine whether developmental environments buffer, filter or redirect behavioural variation.

## Author Contributions

**Clint D. Kelly:** data curation (equal), formal analysis (equal), funding acquisition (lead), methodology (lead), project administration (lead), resources (lead), supervision (lead), writing – original draft (lead), writing – review and editing (lead). **Jules Vaillancourt:** data curation (supporting), investigation (supporting). **Clara Smythe:** investigation (supporting). **Céline Ozkan:** data curation (supporting), investigation (supporting), methodology (supporting).

## Funding

This work was supported by the Natural Sciences and Engineering Research Council of Canada.

## Conflicts of Interest

The authors declare no conflicts of interest.

## Supporting information


**Figure S1:** Posterior predictive check for survival model. The dark line shows the mean number of survivors observed in the data, and the light blue distribution shows the distribution of means from 1000 posterior predictive samples. Close alignment indicates the beta‐binomial model captures the central tendency of the survival counts.
**Figure S2:** Posterior predictive check for distance travelled. The observed data distribution (dark line) is overlaid with distributions from 100 posterior samples (light lines). The model broadly reproduces the location and spread of the observed data.
**Figure S3:** Posterior predictive check for centre use. The observed data distribution (dark line) is overlaid with distributions from 100 posterior samples (light lines). The model broadly reproduces the location and spread of the observed data, although it does not fully reproduce the detailed shape of the observed distribution.
**Figure S4:** Posterior predicted trajectories for distance travelled and centre use. Shaded areas represent 95% credible intervals. Trajectories illustrate how mean behaviour changes across trials for each diet and group‐size combination.
**Figure S5:** Repeated measures of distance travelled across six open‐field trials in adult female earwigs reared under two developmental diet treatments (standard and nutrient‐diluted) and three group‐size treatments (one, four, or eight nymphs per dish). Grey lines connect repeated observations from the same individual; points show individual trial observations. Black lines show treatment‐specific means across trials. Distance travelled was log‐transformed and standardised before plotting.
**Figure S6:** Repeated measures of centre use across six open‐field trials in adult female earwigs reared under two developmental diet treatments (standard and nutrient‐diluted) and three group‐size treatments (one, four, or eight nymphs per dish). Grey lines connect repeated observations from the same individual; points show individual trial observations. Black lines show treatment‐specific means across trials. Centre use was log‐transformed and standardised before plotting.
**Table S1:** Median and interquartile ranges (IQR, 25th–75th percentiles) of distance travelled and centre use by developmental diet and group‐size treatment. Summaries are computed across all six trials and 159 individuals (954 total observations).

## Data Availability

Data and code are available through the OSF repository at https://osf.io/8vmcn/overview?view_only=8a004ba840ed4f3897f3264a74ce0ab2.
