# Supplementary material for: Limited Evidence That Developmental Diet and Density Affect Adult Female Earwig Behaviour Despite Repeatable Individual Differences
Source: Ecol Evol. 2026 Sep 2;16(9):e74325. doi: 10.1002/ece3.74325 (PMC13539217; doi:10.1002/ece3.74325)
Supplement: Supplementary file 1 [file ECE3-16-e74325-s001.docx]

Supplemental Information: Limited evidence that developmental diet and density affect adult female earwig behaviour despite repeatable individual differences

Clint D. Kelly^1,✉^, Jules Vaillancourt^1^, Clara Smythe^2^, and Céline Ozkan^3^

^1^ Département des Sciences biologiques, Université du Québec à Montréal, Montréal, Canada
^2^ Département de biologie, chimie et géographie, Université du Québec à Rimouski, Rimouski, Canada
^3^ Université Claude Bernard Lyon 1, LBBE, UMR 5558, CNRS, VetAgro Sup, Villeurbanne 69622, France

^✉^ Correspondence: [Clint D. Kelly <kelly.clint@uqam.ca>](mailto:kelly.clint@uqam.ca)

# Posterior predictive checks

## Survival model

Posterior predictive checks for the survival model assess whether the beta-binomial model adequately captures the distribution and potential overdispersion in the observed dish-level survival counts.

Figure S 1: Posterior predictive check for survival model. The dark line shows the mean number of survivors observed in the data, and the light blue distribution shows the distribution of means from 1000 posterior predictive samples. Close alignment indicates the beta-binomial model captures the central tendency of the survival counts.

## Behavioural models

Figure S 2: Posterior predictive check for distance travelled. The observed data distribution (dark line) is overlaid with distributions from 100 posterior samples (light lines). The model broadly reproduces the location and spread of the observed data.

Figure S 3: Posterior predictive check for centre use. The observed data distribution (dark line) is overlaid with distributions from 100 posterior samples (light lines). The model broadly reproduces the location and spread of the observed data, although it does not fully reproduce the detailed shape of the observed distribution.

# Posterior predicted trajectories

To address the difficulty of interpreting coefficients from a model with a three-way interaction, we present the posterior predicted trajectories for both behavioural responses across all six diet $\times$ group-size combinations. These plots show the marginal mean trajectories (solid lines) and the 95% credible intervals (shaded regions) for distance travelled and centre use. These trajectories demonstrate that while there are no simple, consistent directional patterns across all treatments, the predicted response to repeated trials varies by diet and group size.

Figure S 4: Posterior predicted trajectories for distance travelled and centre use. Shaded areas represent 95% credible intervals. Trajectories illustrate how mean behaviour changes across trials for each diet and group-size combination.

Figure S 5: Repeated measures of distance travelled across six open-field trials in adult female earwigs reared under two developmental diet treatments (standard and nutrient-diluted) and three group-size treatments (one, four, or eight nymphs per dish). Grey lines connect repeated observations from the same individual; points show individual trial observations. Black lines show treatment-specific means across trials. Distance travelled was log-transformed and standardized before plotting.

Figure S 6: Repeated measures of centre use across six open-field trials in adult female earwigs reared under two developmental diet treatments (standard and nutrient-diluted) and three group-size treatments (one, four, or eight nymphs per dish). Grey lines connect repeated observations from the same individual; points show individual trial observations. Black lines show treatment-specific means across trials. Centre use was log-transformed and standardized before plotting.

# Raw-scale summaries of behavioural responses

Because the primary analysis reports coefficients on standardized log scales, we provide median and interquartile-range summaries of distance travelled and centre use on their original measurement scales (centimetres and seconds, respectively). These are computed across all trials and individuals, stratified by developmental diet and group-size treatment and shown alongside overall summary statistics.

Table S 1: Median and interquartile ranges (IQR, 25th–75th percentiles) of distance travelled and centre use by developmental diet and group-size treatment. Summaries are computed across all six trials and 159 individuals (954 total observations).

| Diet | Density | Observations | Distance (cm) | Centre use (s) |
| --- | --- | --- | --- | --- |
| Standard | 1 nymph | 180 | 113.3 [38.8–258.0] | 48.5 [13.7–174.0] |
| Standard | 4 nymphs | 150 | 112.5 [40.5–203.1] | 39.0 [11.4–204.2] |
| Standard | 8 nymphs | 222 | 115.7 [41.7–246.3] | 75.2 [17.2–213.6] |
| Nutrient-diluted | 1 nymph | 132 | 147.4 [59.7–267.0] | 40.5 [11.4–167.9] |
| Nutrient-diluted | 4 nymphs | 132 | 77.8 [37.0–160.9] | 68.1 [13.6–308.8] |
| Nutrient-diluted | 8 nymphs | 138 | 92.5 [37.7–241.7] | 89.4 [18.6–274.3] |
| Overall | — | 954 | 111.0 [41.5–242.0] | 58.3 [14.0–211.6] |
